# Neurovascular damage in experimental allergic encephalomyelitis: a target for pharmacological control

**DOI:** 10.1080/09629359791415

**Published:** 1997-12

**Authors:** C. Bolton

**Affiliations:** Pharmacology Group School of Pharmacy and Pharmacology University of Bath Bath BA2 7AY UK

## Abstract

The blood-brain barrier (BBB) is composed of a continuous endothelial layer with pericytes and astrocytes in close proximity to offer homeostatic control to the neurovasculature. The human demyelinating disease multiple sclerosis and the animal counterpart experimental allergic encephalomyelitis (EAE) are characterized by enhanced permeability of the BBB facilitating oedema formation and recruitment of systemically derived inflammatory-type cells into target tissues to mediate eventual myelin loss and neuronal dysfunction. EAE is considered a useful model for examining the pathology which culminates in loss of BBB integrity and the disease is now proving valuable in assessing compounds for efficacy in limiting damage at neurovascular sites. The precise mechanisms culminating in EAE-induced BBB breakdown are unclear although several potentially disruptive mediators have been implicated and have been previously identified as potent effectors of cerebrovascular damage in non-disease related conditions of the central nervous system. The review considers evidence that common mechanisms may mediate cerebrovascular permeability changes irrespective of the initial insult and discusses therapeutic approaches for the control of BBB leakage in the demyelinating diseases.


Mediators of Inflammation, 6, 295–302 (1997)

### Neurovascular damage in experimental allergic encephalomyelitis: a target for pharmacological control

C. Bolton

Pharmacology Group, School of Pharmacy and Pharmacology, University of Bath, Bath BA2 7AY, UK
Fax: 01225 826114
Email: c.bolton@bath.ac.uk

**The blood-brain barrier (BBB) is composed of a continuous endothelial layer with pericytes and astrocytes in close proximity to offer homeostatic control to the neurovasculature. The human demyelinating disease multiple sclerosis and the animal counterpart experimental allergic encephalomyelitis (EAE) are characterized by enhanced permeability of the BBB facilitating oedema formation and recruitment of systemically derived inflammatory-type cells into target tissues to mediate eventual myelin loss and neuronal dysfunction. EAE is considered a useful model for examining the pathology which culminates in loss of BBB integrity and the disease is now proving valuable in assessing compounds for efficacy in limiting damage at neurovascular sites. The precise mechanisms culminating in EAE-induced BBB breakdown are unclear although several potentially disruptive mediators have been implicated and have been previously identified as potent effectors of cerebrovascular damage in non-disease related conditions of the central nervous system. The review considers evidence that common mechanisms may mediate cerebrovascular permeability changes irrespective of the initial insult and discusses therapeutic approaches for the control of BBB leakage in the demyelinating diseases.**

**Key words:** Blood-brain barrier, Glucocorticoids, Cyclosporin, N-methyl-D-aspartate receptor, MK-801, Polyamines

### Characteristic Features of the Blood-Brain Barrier

The neurovasculature of the brain and spinal cord originates from invading non-cerebral-derived capillary cells which form a thin continuous endothelial layer devoid of fenestrations and pinocytic vesicles separating blood from the central nervous system (CNS).[1] In close association with the endothelium are pericytes which control endothelial cell proliferation, regulate vessel contractility and synthesize and secrete a variety of vasoactive compounds.[2] Typically, pericytes are polymorphic, elongated multibranched cells that envelope endothelial cells in the microvasculature. Pericyte contractility originates from the presence of both smooth muscle and non-smooth muscle isoforms of actin and myosin. Interestingly, the degree of pericyte contraction may either exacerbate or restrict vessel leakage indicating the cell has a strong influence over blood-brain barrier (BBB) permeability.

On the abluminal surface of the microvessels are situated the astrocytes with end feet in close proximity to the endothelium offering physical support and maintenance of cell function.[3] One particular morphological characteristic of the BBB is the presence of tight junctions which form the physical link between endothelial cells and prevent the non-specific passage of molecules into CNS tissues.[4] Tight junctions form a complex branching network and together with a relatively large density of pericytes, provide an electrical resistance of about $2 \times 10^3$ Ohm/cm$^2$ with low ionic and hydrophilic non-electrolyte permeability.[5,6] Other cellular membranes with similar resistance values are located in the skin, bladder and gastric epithelium. The selective permeability of the endothelium may be a consequence of the abundant mitochrondrial population which provides a high metabolic activity within the constituent cells.[7] The enhanced metabolic state of the BBB is also reflected in the amount of enzymic activity with high concentrations of sodium-potassium ATP-ase, γ-glutamyl transpetidase, alkaline phosphatase and butyryl cholinesterase being observed by histochemical analysis.[8–10]

© 1997 Rapid Science Publishers 

Nutrient uptake into the brain is determined by the lipid solubility of compounds with hydrophilic substances readily traversing the BBB.[11] However, the apparent unrestricted entry of some lipophilic substances can be controlled by a family of intrinsic energy-dependent, membrane-associated proteins which also prevent the transport of highly toxic substances into cerebral tissues.[12] Essential non-lipid soluble nutrients, such as glucose and certain amino acids, must be transported across the neurovasculature through binding to specific membrane-bound proteins.[13] The BBB can also metabolically control the entry of precursor substances, which are derivatised and retained in cerebroendothelial tissue.[14]

Clearly the normally restrictive BBB possesses vital properties which closely regulate the passage of essential and non-essential substances into and out of the CNS. Optimal control of transport mechanisms is of primary importance in maintaining neuronal function and typical cerebral homeostasis. However, a breakdown in normal barrier integrity can occur in many pathological conditions of the CNS such as tumour development, hypertension and ischaemia, to cause increased intercellular leakage at malfunctioning tight junctions and alterations in transport mechanisms.[15]

The human demyelinating disease multiple sclerosis (MS) is characterized by episodic malfunction of the BBB which allows oedema formation and inflammatory cell invasion of CNS tissues.[16] The inducible animal counterpart experimental allergic encephalomyelitis (EAE) also displays neurovascular disruption as a prominent pathological feature.[17] The purpose of this article is to consider the mechanisms and consequences of EAE-induced immune-mediated insults on the BBB. In particular, attention will focus on how resulting damage can be measured and minimized through pharmacological intervention.

### Immunologically Induced Events Mediating BBB Breakdown in Experimental Models of MS

The traditional view of the CNS being an immunoprivileged site originates from an absence of a true lymphatic system and the presence of only minimal numbers of immunocompetent cells, reduced antigen determinant expression and low residual production of immunological mediators.[18] Improvements in methods to monitor cell trafficking have provided evidence that brain and spinal tissues are routinely surveyed by consistent numbers of apparently non-specifically activated T lymphocytes. Facilitated entry of immunocompetent T cells into CNS tissue is closely regulated by the BBB and, in particular, the luminal expression of lymphocyte-directed intracellular adhesion molecules.[19,20]

One CNS disease which is strongly influenced by events at neurovascular sites is the autoimmune condition EAE, a highly reproducible but genetically restricted model of human demyelinating disease.[21] The pathogenesis of EAE is complex and offers many locations at which the disease may be limited. The BBB can be regarded as one such strategic site for regulation and possible control of the disease. However, and despite the importance of events at the BBB in determining the occurrence of EAE, only limited information is available concerning mediators which disrupt the cerebral endothelium and allow CNS access to inflammatory-type cells causing, in the more chronic forms, demyelination and neuronal dysfunction.

Vasoactive amines have been regarded as possible mediators of neurovascular disruption in EAE.[22] Histamine, released from predominant numbers of systemic mast cells during EAE, is present in excess during early neurological disease and has been shown to increase pinocytic vesicle activity in neurovascular isolates.[23,24] Interestingly, use of the vasoactive amine antagonist cyproheptidine can limit the neurological and histological expression of EAE but the drug's direct effects on the immunocompromised BBB is not known.[25] Differentially increased prostaglandin levels have been recorded in CNS tissues from animals with the acute and chronic-relapsing forms of EAE[26,27] and also in the cerebrospinal fluid from MS patients.[28] Prostaglandins of the E and F series have profound regulatory effects on vascular contractility and therefore could substantially alter neurovascular function prior to and during the expression of disease. However, treatment with non-steroidal anti-inflammatory drugs, such as indomethacin, does not prevent the development of EAE and may intensify disease expression.[29,30] The inflammatory cell-derived cytokines interleukin-1 (IL-1) and tumour necrosis factor-$\alpha$ (TNF-$\alpha$) have been strongly implicated in the induction of EAE and, in particular, as mediators of BBB disruption.[31–34] TNF-$\alpha$ levels have also been found to correlate closely with neurovascular disturbances in MS patients experiencing active disease.[35] Interestingly, *in vitro* studies have also shown IL-1 and TNF-$\alpha$ to possess permeability-inducing properties in CNS-derived neuroendothelial preparations.[31,32]

Previous investigations by us have suggested a role for the vasodilatory molecule nitric oxide (NO) in mediating deleterious changes at the BBB[35] although pharmacological inhibition of NO production, in an attempt to control EAE, has provided conflicting results.[36–39] Nevertheless, high levels of NO are present in EAE-diseased CNS tissues providing the potential to cause BBB damage. In associated preliminary studies we have highlighted the importance of the cellular and vasodisruptive polyamines in EAE[40,41] which complements earlier work illustrating the importance of the compounds in non-immune-mediated CNS diseases.[42,43] Both NO and the polyamines can be generated following activation of the N-methyl-D-aspartate (NMDA) receptor located at neuronal and cerebrovascular sites.[44–46] Again, our studies have shown that pharmacological antagonism of the receptor can prevent BBB breakdown,[47] reduce polyamine levels[48] and, as shown by others,[49] the neurological symptoms of EAE.

### The Pathology and Consequences of BBB Breakdown in EAE

The neurovasculature of animals with EAE shows increased numbers of pinocytic vesicles apparently occurring as a result of alterations in energy-dependent processes which regulate transport mechanisms at the BBB.[50,51] Metabolic changes at the BBB are further indicated by a marked reduction in the mitochondrial content of the endothelium at the height of disease.[51,52] Sodium-potassium ATPase activity is upregulated which may have consequences on cellular osmotic pressure and fluid uptake into CNS tissues.[53] Accumulation of oedematous fluid within CNS tissues together with insoluble deposits around nerves can lead to neuronal dysfunction and neurological deficits in EAE.[21,54] In addition to the development of CNS oedema during EAE the parenchymal spaces become characteristically infiltrated by cells of the lymphocyte-macrophage series. Inflammatory products including degradative proteases and soluble immune factors are released thus ensuring continual recruitment and disruption of target tissues.[55,56]

### Techniques to Assess BBB Integrity

Over 40 years have elapsed since the first qualitative experiments on BBB permeability in EAE were undertaken using trypan blue to expose abnormal extravasation at sites of inflammatory cell infiltration.[57] The subsequent use of radiolabelled proteins and autoradiographs of CNS tissues demonstrated vascular disturbances occurred concurrently with initial symptoms of disease.[58,59] Immunohistochemical techniques by Oldstone and Dixon,[60] monitoring leakage of serum fibrinogen, β1C globulin and IgG into CNS tissues, also revealed cerebral vessel abnormalities prior to inflammatory cell infiltration and neurological signs although subsequent quantitative radioisotope methods failed to distinguish between barrier leakage and the occurrence of lesions.[61] Reasons for the discrepancies in the findings are unclear but may be due to a variety of factors including species differences, sample selection and relative sensitivities of techniques employed to detect the pathological changes. Nevertheless, an interval between the passage of solutes across the BBB and the movement of inflammatory cells into the perivasculature would be expected to occur.

Electron microscopic studies have revealed structural alterations at neurovascular sites during the development of EAE which led to the suggestion of leakage through interendothelial tight junctions as a mechanism to enhance BBB permeability.[62,63] Subsequent investigations have failed to confirm the passage of substances via interendothelial routes. Indeed, studies in chronic-relapsing EAE, using magnetic resonance imaging (MRI) scanning together with histological evaluation, have found no evidence of tight junction opening during obvious BBB malfunction.[64] MRI scanning, with the use of gadolinium-linked contrast agents, has been used with great effectiveness in EAE to demonstrate physiological and metabolic changes in the neurovasculature and show that energy-dependent processes and active transport mechanisms are altered during BBB disruption.[65–67]

A particular advantage of the MRI technique is the ability to evaluate ongoing EAE disturbances in individual animals during the course of acute and chronic-relapsing disease. Serial scanning of lesions has revealed barrier disturbances remain for more than a month although shorter times of 1–2 weeks is typical.[68–71] The technique has also been used to confirm a relationship between the severity of BBB disturbances and the specificity and numbers of immunocompetent T lymphocytes required to induce EAE.[72] Moreover, MRI has allowed a detailed comparison between BBB changes in EAE and during MS, at a structural level, which has strengthened the relevance of the animal model in the study of human demyelinating diseases.

Ultrastructural studies of the BBB, prior to MRI, were undertaken using the enzymic tracer horseradish peroxidase[73,74] later to prove useful in establishing the mechanisms involved in loss of neurovascular integrity during acute and chronic-relapsing EAE.[75,76] In particular, the marker was observed in transcytotic vesicles and areas of inflammatory cell infiltration in both forms of disease. Closer observation revealed a large number of vesicles together with tubular structures at parajunctional regions facilitating passage of the tracer across the endothelial cells rather than through tight junctions. Studies by Vorbrodt[77] have used endogenous plasma albumin in conjunction with immunogold cytochemistry to observe the functional state of the BBB at the ultrastructural level. The technique allows various routes of transendothelial or transvascular passage to be studied with a quantitative evaluation of barrier dysfunction.

Use of the inert lipophobic compound mannitol to measure BBB disturbances has also been described[78] and used in EAE.[79,80] Mannitol has no carrier system and diffuses slowly across the intact neurovasculature thus ensuring that any increased passage of the molecule is due to changes in BBB permeability. Neurovascular leakage of radiolabelled mannitol precedes the symptoms of EAE with both parameters correlating well approximately 14 days post-inoculation. Later studies by Lam[17] showed that larger molecular weight substances, such as insulin and albumin, could not detect early changes in barrier function during pre-neurological EAE clearly illustrating the need for caution in the selection of markers for determining BBB abnormalities.

Our studies have acknowledged the earlier investigations of Leibowitz and Kennedy[61] by utilizing radiolabelled albumin in conjunction with a second radioactive marker to quantitate neurovascular breakdown.[81] More recently we have employed a radiolabelled marker, specific to inflammatory-type cells, to accurately quantitate BBB disruption in EAE.[82] In brief, the procedure utilizes the binding of a synthetic tuftsin antagonist to the receptor site expressed on systemically-activated cells which eventually breach the BBB and thereby provide an estimate of leakage at neurovascular targets. Both techniques detect similar alterations in cerebrovascular permeability throughout the course of EAE and the latter system also allows the potential for $\gamma$-camera imaging of disrupted neuroendothelial sites. The development and established use of highly reproducible and refined techniques to measure BBB disturbances can guarantee a confident evaluation of drug effects on preventing or restoring normal neurovascular function in a variety of CNS conditions. However, of the numerous compounds administered in models of EAE only very few have been assessed for direct effects on the BBB.

### Pharmacological Control of Abnormal BBB Permeability

Pathological conditions affecting the CNS often feature BBB breakdown caused by the classic components of acute and chronic inflammation including histamine, arachidonate metabolites, bradykinin and free radicals.[83] Many of these inflammatory mediators can normally be limited by anti-inflammatory-type drugs which curiously often prove inactive at neurovascular sites. However, one group of compounds to unequivocally suppress enhanced permeability at cerebrovascular sites are the glucocorticoids. Original studies by Long and Holladay[84] demonstrated the homeostatic influence of endogenous corticosteroids on the BBB and suggested permeability was closely governed by the hypothalmic-pituitary-adrenal axis. Subsequent investigations by Hedley-Whyte and Hsu[85] and Zylan *et al.*[86] in healthy rodents showed that administration of the synthetic corticoid, dexamethasone (Dex), could reduce permeability below normal levels for a variety of circulating tracers. Interestingly, BBB permeability is enhanced following adrenalectomy and restored to normal by Dex treatment clearly demonstrating a crucial role for the glucocorticoids in maintaining integrity at the neurovasculature. Several studies have detailed the importance of the glucocorticoids in controlling EAE[87–89] and more recent work by us has described the corrective influence of Dex on BBB breakdown when administered therapeutically.[81] The broad effects produced following glucocorticoid administration does not allow a precise evaluation of their actions at neurovascular sites. However, *in vivo* and *in vitro* studies have shown the compounds do have the potential to act directly to limit dysfunction on neuroendothelial cells and associated targets.[90,91]

An alternative, receptor-based, approach to the control of cerebrovascular damage in EAE was first offered through the studies of Brosnan *et al.*[92] and Goldmuntz *et al.*[93] who used the $\alpha$1-adrenergic antagonist prazosin to significantly improve BBB function and also reduce the histological signs and symptoms of disease. The possibility of receptor-mediated events occurring in the loss of BBB integrity during EAE has been more recently considered in our studies using antagonists of the NMDA receptor situated at neuronal and neurovascular locations.[47] The NMDA receptor antagonist MK-801, which gates the open ion channel, dramatically curtails BBB leakage using prophylactic and therapeutic dosing regimes. Additional preliminary studies have revealed that increased CNS polyamine levels during EAE can be significantly reduced following MK-801 treatment strongly implicating a role for these agents in the loss of cerebrovascular integrity.[41,48] Moreover, antagonism of the polyamine site on the NMDA receptor, through the use of the selective non-competitive neuroprotective antagonist ifenprodil, has proved valuable in initial studies to limit neuroendothelial breakdown.[48] We envisage ongoing work with specific antagonists will assist in identifying the subtypes of NMDA receptor and receptor sites involved in mediating BBB breakdown and may lead to the design of new drugs to control cerebrovascular damage.

Another compound with diverse effects is the immunosuppressant cyclosporin A (CsA) which has been used repeatedly to delay the onset and progression of EAE and, in particular, BBB breakdown in selected areas of the CNS.[81,94,95] The drug can influence abnormal cerebrovascular permeability during EAE by limiting the local production of disruptive mediators. For example, CsA has been found to reduce NO generation from vascular preparations and bind to cytosolic proteins involved in the production of neuroendothelial membrane disruptive polyamines.[96–98] However, unlike the glucocorticoids and despite a high lipophilicity, CsA does not readily accumulate in the CNS parenchyma thus reducing pharmacological effects at CNS targets. One reason for the low penetration of cerebral tissues by CsA is the high affinity of the drug for p-glycoprotein, a transmembrane efflux transporter located on the luminal membrane of neuroendothelial cells, which actively pumps

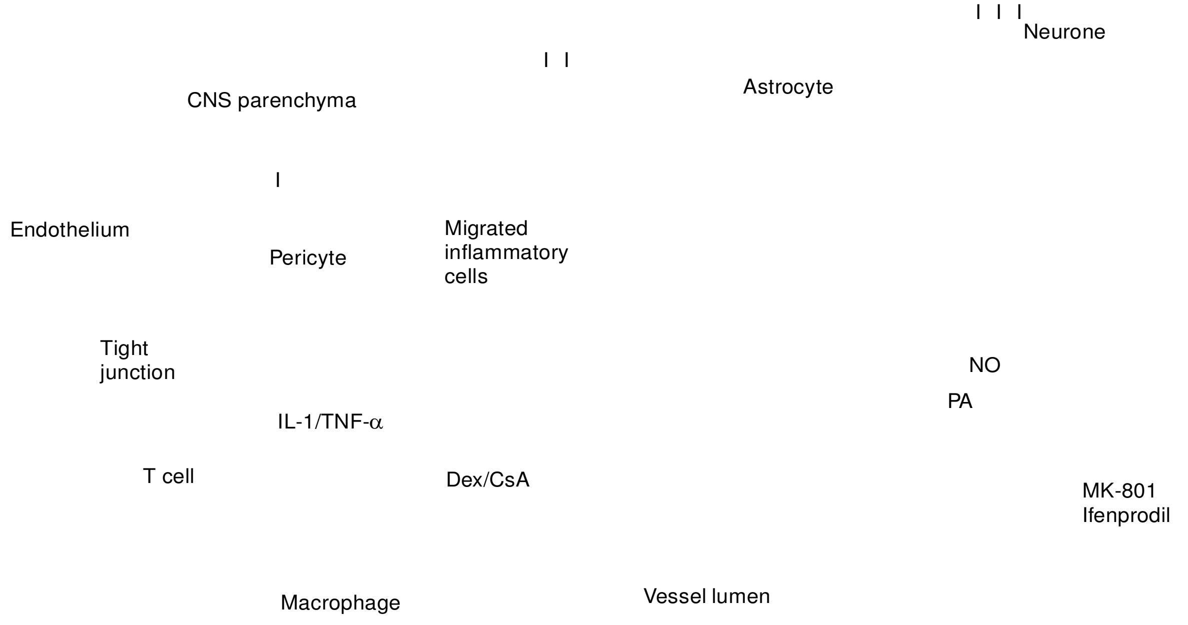

I — Release of mediators, including interleukin-1 (IL-1) and tumour necrosis factor-α (TNF-α), from neuroantigen-activated inflammatory cells to instigate BBB disruption.

I I — Migration of inflammatory cells into CNS parenchyma with passage of plasma constituents across leaky neurovasculature.

I I I — Perpetuation of BBB damage by release of permeability inducing factors including nitric oxide (NO) and polyamines (PA).

Dex — dexamethasone

CsA — cyclosporin A

(receptor symbols) — Activation of receptors on BBB and neurones.

⇐ — Sites of drug action.

FIG. 1. Major events mediating neuroimmune-directed BBB breakdown which may act as sites for drug intervention.

lipophilic molecules back into the circulation.[99–101] Inactivation of the p-glycoprotein pump, through the use of cytotoxic or non-immunosuppressive compounds, has been described[102,103] raising the possibility that drug delivery across the neurovasculature and into the CNS could be enhanced by specific pre-treatment regimes.

### Summary

The use of EAE as a prototype for the human demyelinating condition MS has obvious limitations but one prominent and characteristic feature apparent in both diseases is a sustained loss of BBB integrity. Although the precise mechanisms of neurovascular breakdown in EAE and MS are unknown, similar morphological and immunological-associated changes do occur. In non-disease related conditions of the CNS a sequence of receptor-linked occurrences appear to regulate activation of important biochemical pathways which culminate in extensive neurovascular damage with eventual disruption of neuronal function. Improvements in the design and use of detection systems to monitor BBB breakdown together with analysis of target tissues for possible mediators of cerebrovascular leakage tentatively indicate similar mechanisms may exist in the pathogenesis of EAE and also MS (Fig. 1). Therefore, it is intriguing to speculate that a common series of events occur to cause abnormal BBB permeability irrespective of the initial insult to the neurovasculature. Pharmacological intervention with site-specific drugs may help to clarify the pathways involved in neuroimmune-mediated BBB breakdown and eventually offer therapies to control an abberant and dominant aspect central to the pathology of the demyelinating diseases.

### References

1. Bar T. The vascular system of the cerebral cortex. *Adv Anat Embryo Cell Biol* 1980; **59**: 1–62.
2. Shepro D, Morel NML. Pericyte physiology. *FASEB J* 1993; **7**: 1031–1038.
3. Crone C. The blood-brain barrier: a modified tight epithelium. In: Suckling AJ, Rumsby MG, Bradbury MWB, eds. *The Blood-Brain Barrier in Health and Disease*. Chichester: Ellis Horwood, 1986; 17–40.
4. Goldstein GW, Betz AL. The blood-brain barrier. *Sci Am* 1986; **255**: 70–79.
5. Goldstein GW. Endothelial cell–astrocyte interactions. A cellular model of the blood-brain barrier. *Ann NY Acad Sci* 1988; **529**: 31–39.
6. Engelhardt B, Risau W. Development of the blood-brain barrier. In: Greenwood J, Begley DJ, Segal MB, eds. *New Concepts of a Blood Brain Barrier*. New York: Plenum Press, 1995; 11–31.
7. Cervos-Navarro J, Kannuki S, Nakagawa Y. Blood-brain barrier (BBB). Review from morphological aspect. *Histol Histopath* 1988; **3**: 203–213.
8. Betz AL, Firth JA, Goldstein GW. Polarity of the blood-brain barrier: distribution of enzymes between the luminal and antiluminal membranes of the brain capillary endothelial cells. *Brain Res* 1980; **192**: 17–28.
9. Kreutzberg GW, Tóth L. Enzyme cytochemistry of the cerebral microvessel wall. *Acta Neuropathol* 1983; **8**: 35–41.
10. Joó F. The blood-brain barrier *in vitro*: ten years of research on microvessels isolated from the brain. *Neurochem Int* 1985; **7**: 1–25.
11. Fernstermacher JD, Wei L, Acuff V, *et al.* The dependency of influx across the blood-brain barrier on blood flow and the apparent flow-independence of glucose influx during stress. In: Greenwood J, Begley DJ, Segal MB, eds. *New Concepts of a Blood-Brain Barrier*. New York: Plenum Press, 1995; 89–101.
12. Abbott NJ, Courand PO, Roux F, Begley DJ. Studies on an immortalized brain endothelial cell line: characterisation, permeability and transport. In: Greenwood J, Begley DJ, Segal MB, eds. *New Concepts of a Blood Brain Barrier*. New York: Plenum Press, 1995; 239–249.
13. Bodor N, Brewster M. Problems of delivery of drugs to the brain. *Pharmacol Ther* 1983; **19**: 337–386.
14. Betz AL, Goldstein GW. Specialised properties and solute transport in brain capilliaries. *Ann Rev Physio* 1986; **48**: 142–250.
15. Banks WA, Kastin AJ. Interactions between the blood-brain barrier and endogenous peptides: emerging clinical implications. *Am J Med Sci* 1988; **295**: 459–465.
16. Moor ACE, De Vries HE, De Boer AG, Breimer DD. The blood-brain barrier and multiple sclerosis. *Biochem Pharmacol* 1994; **47**: 1717–1724.
17. Lam DKC. The blood-central nervous system barrier in acute experimental allergic encephalomyelitis. In: Suckling AJ, Rumsby MG, Bradbury MWB, eds. *The Blood-Brain Barrier in Health and Disease*. Chichester: Ellis Horwood, 1986; 17–40.
18. Medawar PB. Immunity to homologous grafted skin III. The fate of skin homografts transplanted to the brain, to subcutaneous tissue, and to the anterior chamber of the eye. *Br J Exp Pathol* 1948; **29**: 58–69.
19. Wilcox CE, Baker D, Butter C, Willoughby DA, Turk JL. Differential expression of guinea pig class II major histocompatibility complex antigens on vascular endothelial cells *in vitro* and in experimental allergic encephalomyelitis. *Cell Immunol* 1989; **120**: 82–91.
20. Fabry Z, Waldschmidt MM, Hendrickson DK, *et al.* Adhesion molecules on murine brain microvascular endothelial cells: expression and regulation of ICAM-1 and Lgp55. *J Neuroimmunol* 1992; **36**: 1–11.
21. Paterson PY. Experimental autoimmune (allergic) encephalomyelitis: induction, pathogenesis and suppression. In: Meischer PA, Mueller-Eberhard HJ, eds. *Textbook of Immunopathology*. New York: Grune & Stratton, 1976; 179–213.
22. Linthicum DS, Munoz JJ, Blaskett A. Acute experimental autoimmune encephalomyelitis in mice. 1. Adjuvant action of *Bordetella pertussis* is due to vasoactive amine sensitisation and increased vascular permeability of the central nervous system. *Cell Immunol* 1982; **73**: 299–310.
23. Dux E, Joó F. Effects of histamine on brain capillaries: fine structural and immunohistochemical studies after intracarotid infusion. *Exp Brain Res* 1982; **47**: 252–258.
24. Orr EL, Stanley NC. Brain and spinal cord levels of histamine in Lewis rats with acute experimental autoimmune encephalomyelitis. *J Neurochem* 1989; **53**: 111–118.
25. Waxman FJ, Taguiam JM, Whitacre CC. Modification of clinical and histopathologic expression of experimental allergic encephalomyelitis by a vasoactive amine antagonist—cyproheptidine. *Cell Immunol* 1984; **85**: 82–93.
26. Bolton C, Gordon D, Turk JL. A longitudinal study of the prostaglandin content of central nervous system tissues from guinea pigs with acute experimental encephalomyelitis. *Int J Immunopharmac* 1984; **6**: 155–161.
27. Bolton C, Parker D, McLeod J, Turk JL. A study of the prostaglandin and thromboxane content of the central nervous tissues with the development of chronic relapsing allergic encephalomyelitis. *J Neuroimmunol* 1986; **10**: 201–208.
28. Bolton C, Turner AM, Turk JL, Prostaglandin levels in cerebrospinal fluid from multiple sclerosis patients in remission and relapse. *J Neuroimmunol* 1984; **6**: 151–159.
29. Bolton C, Cuzner ML. Modification of EAE by non-steroidal anti-inflammatory drugs. In: Davison AN, Cuzner ML, eds. *The Suppression of Experimental Allergic Encephalomyelitis and Multiple Sclerosis*. London: Academic Press, 1980; 189–198.
30. Mannie MD, Pope L, Paterson PY. Indomethacin augments *in vitro* proliferative responses of Lewis rat lymphocytes to myelin basic protein—implications for experimental allergic encephalomyelitis. *Cell Immunol* 1989; **121**: 196–212.
31. Henning B, Goldblum S, McClain C. Interleukin-1 and tumor necrosis factor/cachectin increase endothelial permeability *in vitro*. *J Leukoc Biol* 1987; **42**: 551.
32. Brett J, Gerlach H, Nawroth P, Steinberg S, Godman G, Stern D. Tumour necrosis factor/cachectin increases permeability of endothelial cell monolayers by a mechanism involving regulatory G proteins. *J Exp Med* 1989; **169**: 1977–1991.
33. Kuroda Y, Shimamoto Y. Human tumor necrosis factor-$\alpha$ augments experimental allergic encephalomyelitis in rats. *J Neuroimmunol* 1991; **34**: 159–164.
34. Baker D, O'Neill JK, Turk JL. Cytokines in the central nervous system of mice during chronic relapsing allergic encephalomyelitis. *Cell Immunol* 1991; **134**: 505–510.
35. Sharief MK, Thompson EJ. *In vivo* relationship of tumour necrosis factor-α to blood-brain barrier damage in patients with active multiple sclerosis. *J Neuroimmunol* 1992; **38**: 27–34.
36. Scott GS, Paul C, Whitehouse J, Williams KI, Bolton C. Immunomodulation of nitric oxide (NO) and its contribution to neurovascular permeability during experimental allergic encephalomyelitis (EAE). *Inflamm Res* 1995; **44**: S223.
37. Cross AH, Misko TP, Lin RF, Hickey WF, Trotter JL, Tilton RG. Aminoguanidine, an inhibitor of inducible nitric oxide synthase ameliorates experimental autoimmune encephalomyelitis in SJL mice. *J Clin Invest* 1994; **93**: 2684–2690.
38. Ruuls SR, Van der Linden S, Huitinga I, Dijkstra CD. Nitric oxide as immunesuppressor in experimental allergic encephalomyelitis. *J Neuroimmunol* 1994; **54**: 193.
39. Zielasek JJ, Jung S, Gold R, Liew FY, Toyka KV, Hartung H-P. Administration of nitric oxide synthase inhibitors in experimental autoimmune neuritis and experimental autommune encephalomyelitis. *J Neuroimmunol* 1995; **58**: 81–88.
40. Scott GS, Williams KI, Bolton C. A pharmacological study on the role of nitric oxide in the pathogenesis of experimental allergic encephalomyelitis. *Inflamm Res* 1996; **45**: 524–529.
41. Paul C, Scott GS, Barbour M, Seiler N, Bolton C. N-methyl-D-aspartate (NMDA) receptor-mediated events contribute to neurovascular breakdown during experimental allergic encephalomyelitis (EAE). *Biochem Soc Trans* 1997; **25**: 1665.
42. Koenig H, Goldstone AD, Lu CY. Blood-brain barrier breakdown in brain edema following cold injury is mediated by microvascular polyamines. *Biochem Biophys Res Commun* 1983; **116**: 1039–1048.
43. Koenig H, Goldstone AD, Lu CY. Blood brain barrier breakdown in cold-infused brain is linked to a biphasic stimulation of ornithine decarboxylase activity and polyamine synthesis: both are coordinately inhibited by verapamil, dexamethasone, and aspirin. *J Neurochem* 1989; **52**: 101–109.
44. Garthwaite J, Garthwaite G, Palmer RMJ, Moncada S. NMDA receptor activation induces nitric oxide synthesis from arginine in rat brain slices. *Eur J Pharmacol* 1989; **172**: 413–416.
45. Koenig H, Goldstone AD, Lu CY, Trout JJ. Brain polyamines are controlled by N-methyl-D-aspartate receptors during ischemia and recirculation. *Stroke* 1990; **21**: 98–111.
46. Koenig H, Trout JJ, Goldstone AD, Lu CY. Capillary NMDA receptors regulate blood-brain barrier function and breakdown. *Brain Res* 1992; **588**: 297–303.
47. Bolton C, Paul C. MK-801 limits neurovascular dysfunction in experimental allergic encephalomyelitis. *J Pharm Exp Therap* 1997; **282**: 397–402.
48. Paul C, Seiler NB, Bolton C. Pharmacological evidence of a role for N-methyl-D-aspartate (NMDA) receptor generated polyamines in neurovascular breakdown during experimental allergic encephalomyelitis (EAE). *J Neuroimmunol* (in press).
49. Wallstrom E, Diener P, Ljungdahl A, Khademi M, Nilsson C-G, Olsson T. Memantine abrogates neurological deficits, but not CNS inflammation, in Lewis rat experimental autoimmune encephalomyelitis. *J Neurol Sci* 1996; **137**: 89–96.
50. Claudio L, Kressy Y, Norton WT, Brosnan CF. Increased vesicular transport and decreased mitochondrial content in blood-brain barrier endothelial cells during experimental allergic encephalomyelitis. *Am J Pathol* 1989; **135**: 1157–1168.
51. Hawkins CP, Munro PMG, Landon DN, McDonald WI. Metabolically dependent blood-brain barrier breakdown in chronic relapsing experimental allergic encephalomyelitis. *Acta Neuropathol* 1992; **83**: 630–635.
52. Oldendorf WH, Cornford ME, Brown WJ. The large apparent work capacity of the blood-brain barrier: a study of the mitochondrial content of capillary endothelial cells in brain and other tissues of the rat. *Ann Neurol* 1977; **1**: 409–417.
53. Kato S, Nakamura H. Ultrastructural and ultracytochemical studies on the blood-brain barrier in chronic relapsing experimental allergic encephalomyelitis. *Acta Neuropathol* 1989; **77**: 455–464.
54. Pender MP. Demyelination and neurological signs in experimental allergic encephalomyelitis. *J Neurol* 1987; **15**: 11–24.
55. Nathan CF. Secretory products of macrophages. *J Clin Invest* 1987; **79**: 319–326.
56. Hartung H-P, Jung S, Stoll G, *et al.* Inflammatory mediators in demyelinating disorders of the CNS and PNS. *J Neuroimmunol* 1992; **40**: 197–210.
57. Barlow CF. A study of abnormal blood-brain barrier permeability in experimental allergic encephalomyelitis. *J Neuropathol Exp Neurol* 1956; **15**: 196–208.
58. Vulpe NM, Hawkins A, Rozdilsky B. Permeability of cerebral blood vessels in experimental allergic encephalomyelitis studied by radioactive iodinated bovine albumin. *Neurology* 1960; **10**: 171–177.
59. Cutler RW, Lorenzo AV, Barlow CF. Brain vascular permeability to $^{125}$I gamma globulin and leucocytes in allergic encephalomyelitis. *J Neuropath Exp Neurol* 1967; **26**: 558–571.
60. Oldstone MBA, Dixon FJ. Immunohistochemical study of allergic encephalomyelitis. *Am J Path* 1968; **52**: 251–263.
61. Leibowitz S, Kennedy L. Cerebral vessel permeability and cellular infiltration in experimental allergic encephalomyelitis. *Immunology* 1972; **22**: 859–869.
62. Lampert P, Carpenter S. Electron microscopic studies of the vascular permeability and the mechanism of demyelination in experimental allergic encephalomyelitis. *J Neuropath Exp Neurol* 1965; **24**: 11–24.
63. Hirano A, Dembitzer HM, Becker NH, Levine S, Zimmerman HM. Fine structural alterations of the blood-brain barrier in experimental allergic encephalomyelitis. *J Neuropath Exp Neurol* 1970; **29**: 432–440.
64. Hawkins CP, Munro PMG, Mackenzie F, *et al.* Duration and selectivity of blood-brain barrier breakdown in chronic relapsing experimental allergic encephalomyelitis studied by gadolinium–DTPA and protein markers. *Brain* 1990; **113**: 365–378.
65. Stewart WA, Alvord EC, Hruby S, Hall LD, Paty DW. Early detection of experimental allergic encephalomyelitis by magnetic resonance imaging. *Lancet* 1985; **ii**: 898.
66. Kuharik MA, Edwards MK, Farlow MR, *et al.* Gd-enhanced MR imaging of acute and chronic experimental demyelinating lesions. *Am J Neuroradiol* 1988; **9**: 643–648.
67. Grossman RI, Lisak RP, Macchi PJ, Joseph PM. MR of acute experimental allergic encephalomyelitis. *Am J Neuroradiol* 1987; **8**: 1045–1048.
68. Brown WJ. The capillaries in acute and subacute multiple sclerosis—a morphometric analysis. *Neurology* 1978; **28**: 84–92.
69. Miller DH, Rudge P, Johnson G, *et al.* Serial gadolinium enhanced magnetic resonance imaging in multiple sclerosis. *Brain* 1988; **111**: 927–939.
70. Hawkins CP, Mackenzie F, Tofts P, Du Boulay EPGH, McDonald WI. Patterns of blood-brain barrier breakdown in inflammatory demyelination. *Brain* 1991; **114**: 801–810.
71. Morrisey SP, Stodal H, Zettl U, *et al.* *In vivo* MRI and its histological correlaters in acute adoptive transfer experimental allergic encephalomyelitis. *Brain* 1996; **119**: 239–248.
72. Namer IJ, Steibel J, Poulet P, *et al.* Blood-brain barrier breakdown in MBP-specific T cell induced experimental allergic encephalomyelitis. *Brain* 1993; **116**: 147–149.
73. Reese TS, Karnovsky MSJ. Fine structural localisation of a blood-brain barrier to exogenous peroxidase. *J Cell Biol* 1967; **34**: 207–217.
74. Brightman MW, Reese TS. Junctions between intimately opposed cell membranes in the vertebrate brain. *J Cell Biol* 1969; **40**: 648–677.
75. Claudio L, Kress Y, Norton WT, Brosnan CF. Increased vesicular transport and decreased mitochondrial content in blood-brain barrier endothelial cells during experimental autoimmune encephalomyelitis. *Am J Pathol* 1989; **135**: 1157–1168.
76. Lossinsky AS, Badmajew V, Robson JA, Moretz RC, Wisniewski HM. Sites of egress of inflammatory cells and horseradish peroxidase transport across the blood-brain barrier in a murine model of chronic relapsing experimental allergic encephalomyelitis. *Acta Neuropathol* 1989; **78**: 359–371.
77. Vorbrodt AW. The application of quantitative immunocytochemistry for the evaluation of blood-brain barrier (BBB) to endogenous albumin. In: Greenwood J, Begley DJ, Segal MB, eds. *New Concepts of a Blood-Brain Barrier*. New York: Plenum Press, 1995; 39–46.
78. Zlokovic BV, Lipovac MN, Begley DJ, Dawson H, Rakic L. Slow penetration of thyrotropin releasing hormone across the blood-brain barrier of *in situ* perfused guinea-pig brain. *J Neurochem* 1988; **51**: 252–257.
79. Oldendorf WH, Towner HF. Blood-brain barrier and DNA changes during the evolution of experimental allergic encephalomyelitis. *J Neuropathol Exp Neurol* 1974; **33**: 616–631.
80. Daniel PM, Lam DKC, Pratt OB. Changes in the effectiveness of the blood-brain barrier and blood-spinal cord barrier in experimental allergic encephalomyelitis. *J Neurol Sci* 1981; **52**: 211–219.
81. Paul C, Bolton C. Inhibition of blood-brain barrier disruption in experimental allergic encephalomyelitis by short-term therapy with dexamethasone and cyclosporin A. *Int J Immunopharm* 1995; **17**: 497–503.
82. Peers SH, Paul C, Woodhouse L, Thornback JR, Goodbody AE, Bolton C. The study of $^{99m}$Tc RP128 in experimental allergic encephalomyelitis (EAE), an animal model of multiple sclerosis. *J Nuclear Med* 1997; **38**: 187P.
83. Wahl M, Schilling L, Unterberg A, Baethmann A. Autocoids as mediators of vasogenic brain oedema. In: Greenwood J, Begley DJ, Segal MB, eds. *New Concepts of a Blood-Brain Barrier*. New York: Plenum Press, 1995; 147–148.
84. Long JB, Holladay JW. Blood-brain barrier: endogenous modulation by adrenal-cortical function. *Science* 1985; **227**: 1580–1583.
85. Hedley-Whyte ET, Hsu DW. Effect of dexamethasone on the blood-brain barrier in the normal mouse. *Ann Neurol* 1986; **19**: 373–377.
86. Zlylan YZ, Le Fauconnier UJM, Bernard G, Bourre JM. Effects of dexamethasone on transport of α-aminobutyric acid across the blood-brain barrier. *J Neurochem* 1988; **51**: 1338–1342.
87. Levine S, Sowinski R. Therapy of allergic encephalomyelitis in rats after onset of paralysis. In: Davison AN, Cuzner ML, eds. *The Suppression of Experimental Allergic Encephalomyelitis and Multiple Sclerosis*. London: Academic Press, 1980; 199–209.
88. MacPhee IAM, Antoni FA, Mason DW. Spontaneous recovery of rats from experimental allergic encephalomyelitis is dependent on regulation of the immune system by endogenous adrenal corticosteroids. *J Exp Med* 1989; **169**: 431–445.
89. Bolton C, Flower RJ. The effects of the anti-glucocorticoid RU38486 on steroid-mediated supression of experimental allergic encephalomyelitis in the Lewis rat. *Life Sci* 1989; **45**: 97–104.
90. De Kloet ER, Cousin MA, Veldhuis HD, Voorhuis TD, Lando D. Glucocorticoids modulate the response of ornithine decarboxylase to unilateral removal of the dorsal hippocampus. *Brain Res* 1983; **275**: 91–98.
91. Jung-Testas I, Renoir M, Bugnard H, Greene GL, Baulieu E. Demonstration of steroid hormone receptors and steroid action in primary cultures of rat glial cells. *J Steroid Biochem Mol Biol* 1992; **41**: 621–631.
92. Brosnan CF, Goldmuntz EA, Cammer W, Factor SM, Bloom BR, Norton WT. Prazosin an alpha-1 adrenergic receptor antagonist, suppresses experimental autoimmune encephalomyelitis in the Lewis rat. *Proc Natl Acad Sci USA* 1985; **82**: 5915–5921.
93. Goldmuntz EA, Brosnan CF, Norton WT. Prazosin treatment suppresses increased vascular permeability in both acute and passively transferred experimental autoimmune encephalomyelitis in the Lewis rat. *J Immunol* 1986; **137**: 3444–3450.
94. Rumjanek VM, Smith LA, Morley J. Modulation by cyclosporin-A of mononuclear cell distribution during experimental allergic encephalomyelitis. *Int J Immunopharmacol* 1984; **6**: 99–104.
95. Reiber H, Suckling AJ. Cyclosporin-A treatment of experimental allergic encephalomyelitis: changes in immunological regulation and blood-CSF barrier function. *J Neuroimmunol* 1986; **12**: 121–130.
96. Rego A, Vargas R, Cathapermal S, Kuwahara M, Foegh ML, Ramwell PW. Systemic vascular effects of cyclosporin A treatment in normotensive rats. *J Pharmacol Exp Ther* 1991; **259**: 905–915.
97. Gallego MJ, Farre AL, Riesco A, *et al.* Blockade of endothelium-dependent responses in conscious rats by cyclosporin A: effect of L-arginine. *Am J Physiol Heart Circ Physiol* 1993; **264**: H708–H714.
98. Ryffel B. Cyclosporin binding proteins. Identification, distribution, function and relation to FK binding proteins. *Biochem Pharmacol* 1993; **46**: 1–12.
99. Begley DJ, Squires LK, Zlokovic BV, *et al.* Permeability of the blood-brain barrier to the immunosuppressive cyclic peptide cyclosporin A. *J Neurochem* 1990; **55**: 1222–1230.
100. Wang Q, Yang H, Miller DW, Elmquist WF. Effect of the p-glycoprotein inhibitor, cyclosporin A, on the distribution of rhodamine-123 to the brain: an *in vivo* microdialysis study in freely moving rats. *Biochem Biophys Res Comm* 1995; **211**: 719–726.
101. Sakata A, Tama I, Kawazuk K, *et al. In vivo* evidence for ATP-dependent and P-glycoprotein-mediated transport of cyclosporin A at the blood-brain barrier. *Biochem Pharmacol* 1994; **48**: 1989–1992.
102. Begley DJ, Evans JE. Vincristine in therapeutic doses appears to increase blood-brain permeability to colchicine in the anaesthetised guinea-pig. *J Physiol* 1992; **446**: 449P.
103. Drion M, Lemarie M, Lefauconnier JM, Scherrmann JM. Role of p-glycoprotein in the blood-brain transport of colchicine and vinblastine. *J Neurol* 1996; **67**: 1668–1693.

ACKNOWLEDGEMENTS. The author acknowledges the financial support of the Multiple Sclerosis Society of Great Britain and Northern Ireland and the charity SEARCH who have generously funded our studies on the BBB in EAE. The author is also grateful to Dr Carolyn Paul for useful discussions on aspects of the review.

**Received 15 May 1997; accepted 28 July 1997**
